# Dried Blood Spot (DBS) Methodology Study for Biomarker Discovery in Lysosomal Storage Disease (LSD)

**DOI:** 10.3390/metabo11060382

**Published:** 2021-06-13

**Authors:** Corina-Marcela Rus, Sebastiano Di Bucchianico, Claudia Cozma, Ralf Zimmermann, Peter Bauer

**Affiliations:** 1 Centogene GmbH, Am Strande 7, 18055 Rostock, Germany; Claudia.Cozma@centogene.com (C.C.); Peter.Bauer@centogene.com (P.B.); 2 Institute of Chemistry, University of Rostock, Dr.-Lorenz-Weg 1, 18051 Rostock, Germany; ralf.zimmermann@helmholtz-muenchen.de; 3 Helmholtz Zentrum München, Ingolstädter Landstraße 1, 85764 Neuherberg, Germany; dibucchianico@helmholtz-muenchen.de

**Keywords:** mass spectrometry, metabolomics, biomarkers, lysosomal storage diseases (LSD), dried blood spot (DBS)

## Abstract

Lysosomal storage diseases (LSDs) are a heterogeneous group of inherited metabolic diseases caused by mutations in genes encoding for proteins involved in the lysosomal degradation of macromolecules. They occur in approximately 1 in 5000 live births and pose a lifelong risk. Therefore, to achieve the maximum benefit from LSDs therapies, a fast and early diagnosis of the disease is required. In this framework, biomarker discovery is a significant factor in disease diagnosis and in predicting its outcomes. On the other hand, the dried blood spot (DBS) based metabolomics platform can open up new pathways for studying non-directional hypothesis approaches to biomarker discovery. This study aims to increase the efficiency of the developed methods for biomarker development in the context of rare diseases, with an improved impact on the reliability of the detected compounds. Thereby, we conducted two independent experiments and integrated them into the screening of the human blood metabolome: (1) comparison of EDTA blood and filter cards in terms of their suitability for metabolomics studies; (2) optimization of the extraction method: a side-by-side comparison of a series of buffers to the best utility to the disease of interest. The findings were compared to previous studies across parameters such as metabolite coverage, sample type suitability, and stability. The results indicate that measurements of metabolites are susceptible to differences in pre-analytical conditions and extraction solvents. This proposed approach can increase the positive rate of the future development of biomarkers. Altogether, the procedure can be easily adapted and applied to other studies, where the limited number of samples is a common barrier.

## 1. Introduction

After five decades of comprehensive research on the sample quality in metabolomics study, the criteria needed for quality sampling and their influence on the research outcome are still not resolved to complete satisfaction [1]. Hundreds of scientific publications provide a set of guidelines to help select the sample types for different studies [2]. Since there is no universal approach, a clear understanding of patient samples’ characteristics is essential to select the appropriate sample matrix that derives meaningful findings [3]. Over the past decades, dried blood spot (DBS) technology has become a convenient tool in both qualitative and quantitative laboratory analysis [4]. Its applicability saw a significant expansion in recent decades, with a shift from basic to clinical research and medicine [5]. The advantage of DBS technology over other sampling techniques has been extensively tested and published [6]. There are a number of characteristics that make DBS “easy”: it is easy to prepare, easy to receive, easy to use, and easy to store. These key elements made them the method of choice in research [7].

To date, over 6000 distinct types of rare diseases have been described in the literature [8,9], and the number is updated every year with 250–280 new conditions [10]. Of these, lysosomal storage diseases, also referred to herein as LSDs, are a group of more than 70 different inborn metabolic errors with a combined occurrence of around 1 in 5000 live births [11]. This research focused on LSDs due to their monogenic origins, as the majority of metabolic defects are detectable in the metabolomic profile. Novel analytical technologies are needed [12] to advance knowledge and speed up progress towards treatment options for rare diseases [13]. The field of translational metabolomics helps to boost biomarker development [14]. Biomarkers play a pivotal role in preclinical studies [15] as key indicators that allow for the early diagnosis and monitoring of disease [16,17].

The need for disease-specific biomarkers is high [18], and many putative biomarkers are identified in publications every year [19]. Nonetheless, only a few of them have made their transition from bench to bedside [20], and the approval rate remains too low [21]. Successful biomarker discovery requires extensive research, yet the process is slow [18], and some shortages occur at various stages of discovery [22]. There are several reasons for the shortfall in the biomarker pipeline [23], and lack of standardized methodology was cited as the number one purported reason.

Numerous studies have linked the sample selection and extraction protocols as factors that provide the best outcomes [24]. However, there is no consensus on the optimal experimental design, and the approaches are usually correlated with immediate availability and depend on personal aims, triggering deviating results [25,26]. Here, we report the establishment of a proper stratification of the samples used in metabolomics studies to identify the processing methods that bring accurate results in the field of biomarker discovery. This approach helped to minimize the extraneous variable disunity and reduced the errors of analytical experiments to acquire specificity.

## 2. Results

### 2.1. Stability Study

The stability study was divided into (1) study evaluating the stability of the metabolites in DBS samples; (2) study testing the stability of the metabolites in DBS cards prepared from fresh and frozen blood.

#### 2.1.1. Stability Study 1

##### Storage at −20 °C over an Extended Period Has a Major Impact on Metabolite Stability

**Figure 1:**
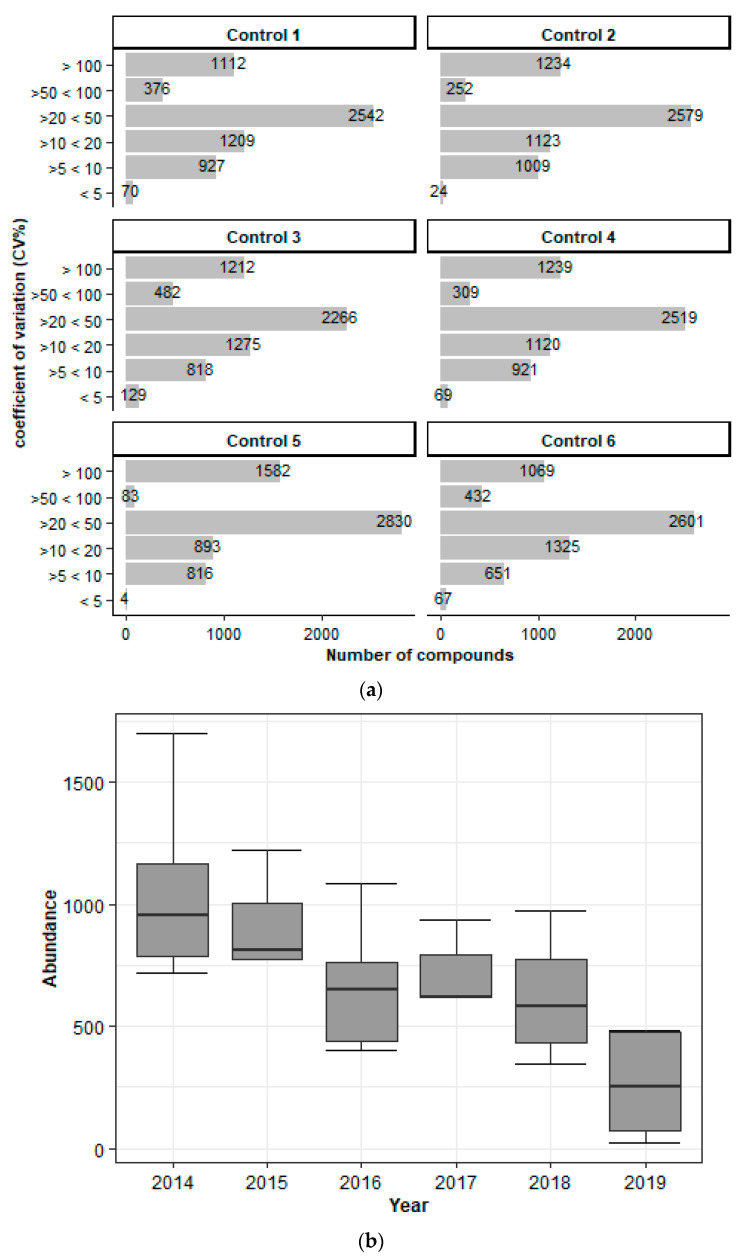
Investigation of the DBS stability. (**a**) The effect of six years of storage on the stability of DBS cards. Per year, blood was drawn from the same individuals (six controls), dripped onto DBS cards within two hours of collection, and stored at −20 °C. As seen in the figure, the optimal variation (5%) was present in few metabolites, while most of them had variations of up to 50%. (**b**) Box plots show an example of a random compound (7.30_439.3416 *m/z*) in DBS samples from the six control subjects collected from 2014 to 2019 and its variation in abundance throughout the years. Box = 25th and 75th percentiles; bars = min and max values.

For the samples used in the stability study, the extraction was carried out using the method described by Cozma et al. 2017 [27]. First, metabolite variation was studied using samples collected from the same individuals (*n* = 6) over six years and stored at −20 °C. Figure 1a shows that a low coefficient of variation (<5%) was present in only 1% of the metabolites, whereas more than 40% of them had a coefficient of variation in the range of 20% to 50%. Additionally, Figure 1b exemplifies the year-to-year variation of the metabolites in DBS stored at −20 °C. These findings indicate that filter cards, even when kept at low temperatures (−20 °C), are unstable from one year to the next.

##### The Stability of the Metabolites Is Affected by Short-Term Storage in Various Conditions

**Figure 2:**
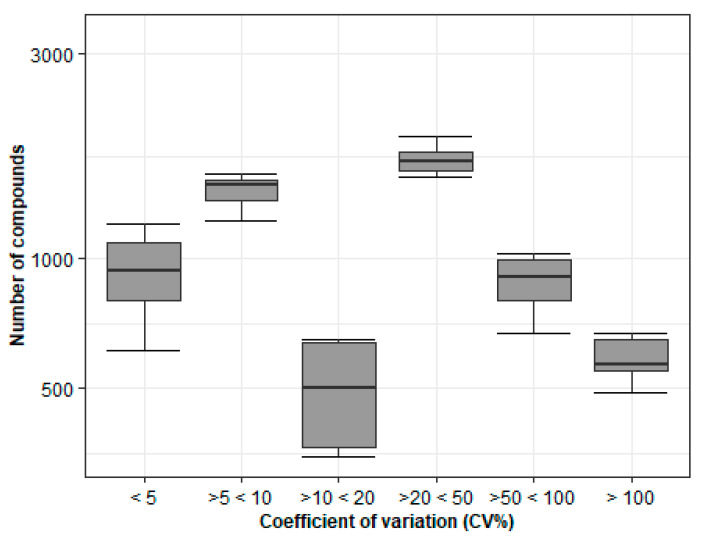
Inter-day influence (three days) of the storage conditions on the human blood metabolome. When DBS samples stored at room temperature were compared to DBS samples maintained at −20 °C, majority of the compounds showed a CV ranging from 20% to 50%.

To validate the prior findings, another test was performed to evaluate the inter-day variation of metabolite yield. This test required the analysis of fresh blood samples stored at RT and −20 °C. Blood was drawn from the same control subjects as in the previous experiment. Following blood collection, duplicate filter cards were prepared. For three days, one duplicate was kept at room temperature (RT), while the other was stored in the freezer (−20 °C). The samples were prepared, extracted, and analyzed in a batch to exclude any deviations. The results showed that three-day storage was sufficient to detect variations in the stability of the analytes when comparing DBS samples stored at RT to those stored at −20 °C (Figure 2).

#### 2.1.2. Stability Study 2

##### Metabolite Yield Is Influenced by Storage Conditions, Sample Types, and Card Age

**Figure 3:**
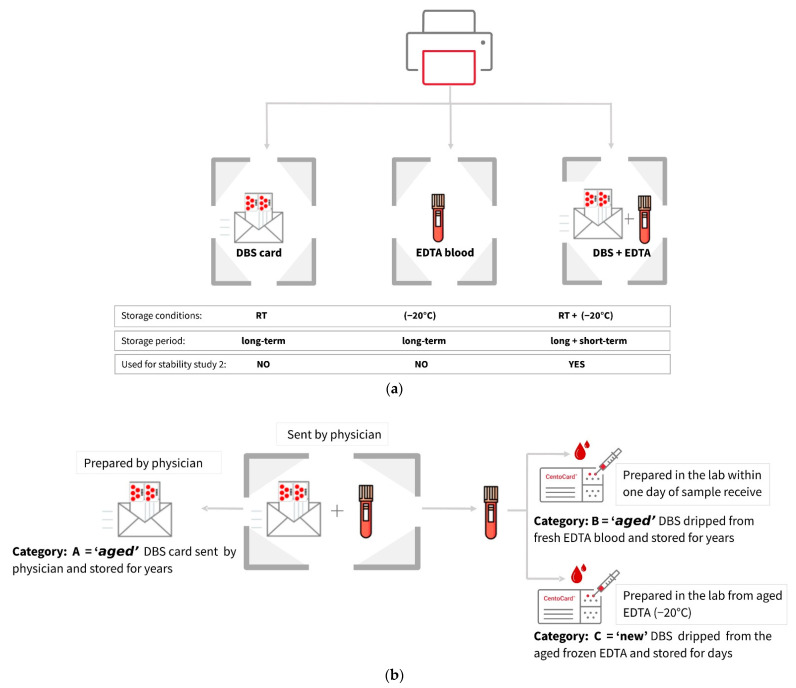
Overview of sample selection, storage and DBS preparation. (**a**) Type of samples received from physician per patient. The samples received from each patient were in the form of either DBS card, or whole EDTA blood, or DBS and EDTA. Stability study 2 comprised only patients with DBS and EDTA samples combined. (**b**) Three different categories of samples were used in stability study 2.

**Figure 4:**
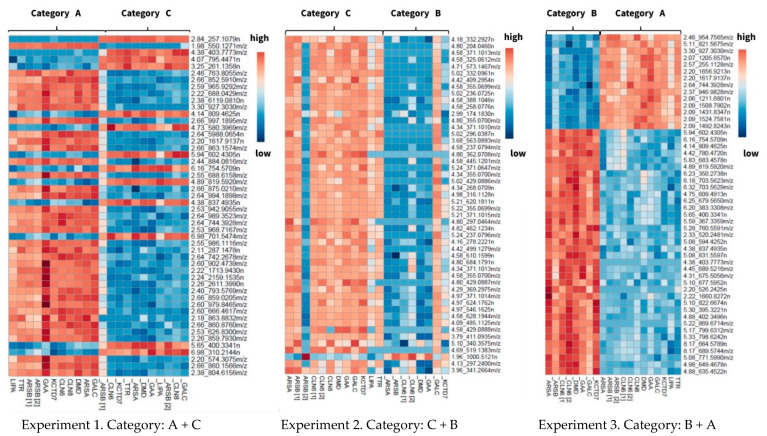
The heatmaps show differences in the abundance of identified compounds across various types of samples. Only the 50 most significant compounds were selected. The colors indicate the abundance of the metabolites: brown indicating the higher level and blue the lower level. The darker the square, the more significant the difference. The data were log-transformed and auto-scaled. The individual samples (columns) and compounds (rows) are separated using hierarchical clustering (Ward’s algorithm).

The next approach was to compare the DBS samples obtained by spotting fresh EDTA whole blood and frozen blood, after long and short-term storage. Here, the cohort comprised subjects with different types of LSDs, and the patients selected were the ones from whom we received both DBS and EDTA whole blood samples for analysis (Figure 3a). From the EDTA blood, the cards were prepared in duplicates at separate points in time. Therefore, one set of DBS cards was prepared within one day of receiving the blood (fresh whole blood), and the other set was prepared after the storage of the EDTA blood at −20 °C for a long period of time (frozen blood). Figure 3b represents a detailed overview of the sample selection and preparation before the analytical study. The results once again revealed a high fluctuation of metabolites (Figure 4). These findings reiterate the importance of sample storage conditions, sample age, and type regarding their suitability for metabolomics studies. These parameters accounted for the differences in the metabolite yield when the assessed batch was made up of heterogeneous samples.

### 2.2. Optimization of Extraction Solvent

#### 2.2.1. The Extraction Solvent Methanol: Acetonitrile Produced Metabolites with the Highest Peak Intensity

Four different buffers were tested and compared for the LC-qTOF/MS-based metabolomics analysis. These were as follows: dimethyl sulfoxide: water, methanol: acetonitrile, isopropanol: acetonitrile: water, and ammonium acetate: water (Table 1a). The data were evaluated based on the total number of metabolites and their abundances.

The raw abundances detected by untargeted MS were normalized in Progenesis using the default parameters. It is important to note that a filter was applied for the selection of compounds. This means that specific values for *m/z*, retention time, charge, fold change, coefficient of variation, and compound abundance were chosen. Only the compounds that passed the filter were considered trustworthy for further investigations.

**Figure 5:**
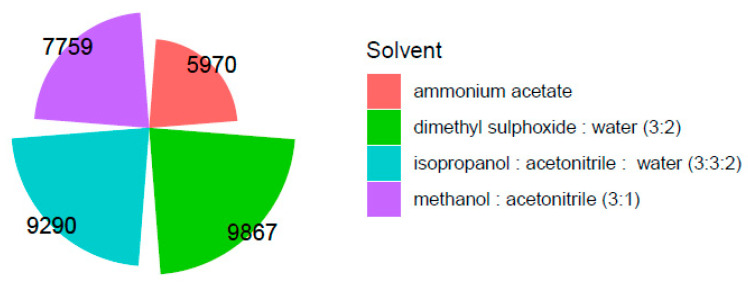
A comparison of the total number of compounds extracted from each of the four extraction solvents.

**Figure 6:**
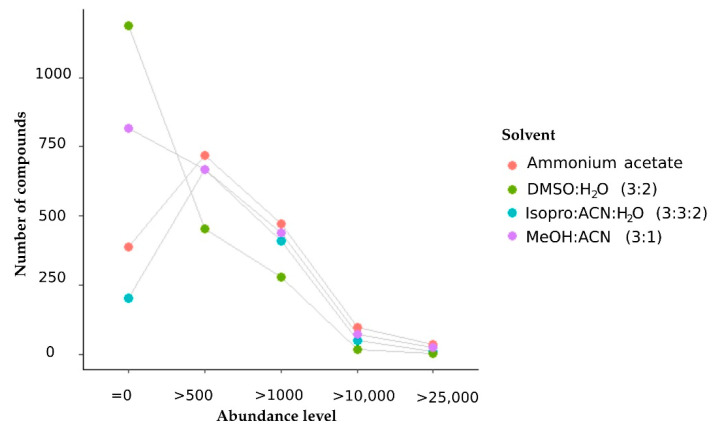
Dotted lines represent the number of features detected and their abundances across the four different extraction solvents. Each dot represents a solvent type.

The results revealed that dimethyl sulfoxide: water was the most effective extraction buffer in terms of metabolome coverage (Figure 5), whereas the mixture of methanol and acetonitrile (3:1, *v/v*) provided the highest number of metabolites with intensities exceeding one thousand (Figure 6).

Extraction with 2 mM ammonium acetate in water (pH = 8) produced metabolites with high concentrations, but the global metabolome coverage was the lowest of the four solvents studied. The isopropanol: acetonitrile: water ranked second in the metabolome coverage, but the number of compounds with high intensity was lower than the other mixtures.

**Figure 7:**
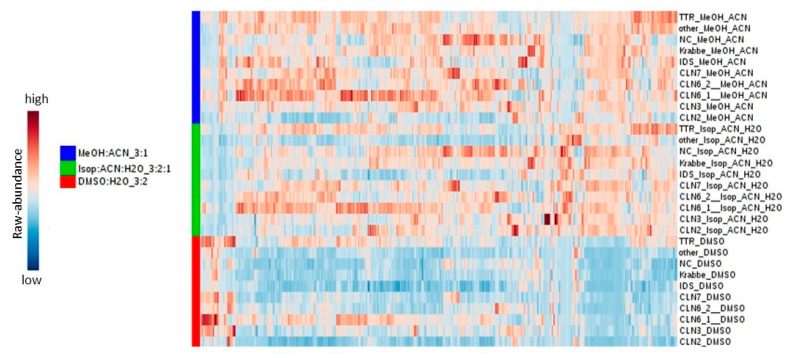
Heatmap showing the reproducibility of the extraction procedure.

**Figure 8:**
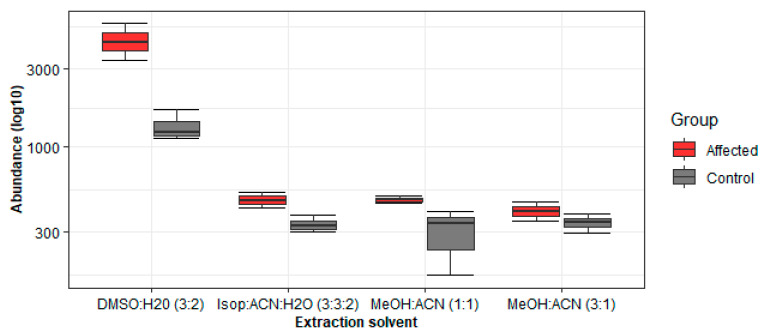
Boxplots showing the distribution of a random metabolite (4.40_421.2811 *m/z*) across different extraction solvents. Control–asymptomatic LSD subjects (*n* = 12); affected–LSD symptomatic patients (*n* = 8). Box = 25th and 75th percentiles; bars = min and max values.

Given the preliminary results, we assume that methanol is a better complement for detecting high-abundance metabolites (Figure 7). Furthermore, this study demonstrated that the extraction solvent for LC-MS-based metabolomics has a visible effect on biomarker discovery projects (Figure 8).

#### 2.2.2. Methanol–Acetonitrile (3:1) Ranks Well in Terms of Metabolome Coverage and Metabolite Intensity

The next step was a comparative study of methanol–acetonitrile (3:1, *v/v*) with other methanol mixtures at various ratios, such as methanol 100%, methanol–water (1:1, *v/v*), and methanol–acetonitrile (1:1, *v/v*). The results were evaluated based on the total number of features and their abundances. Methanol–acetonitrile (3:1, *v/v*) proved to be the most effective mixture for extracting metabolites with high intensity. Table 1a shows the subtle but significant differences among the four different extraction solvents and the comparison of the various methanol mixtures. The considerable overlap between methanol 100% and methanol: acetonitrile (1:1, *v/v*), Table 1b, is noteworthy in terms of metabolome coverage and the number of features with high intensity.

#### 2.2.3. The Application of Learned Principles to the CLN6 Metabolomics Study Helps in the Discovery of Disease-Specific Metabolites

**Figure 9:**
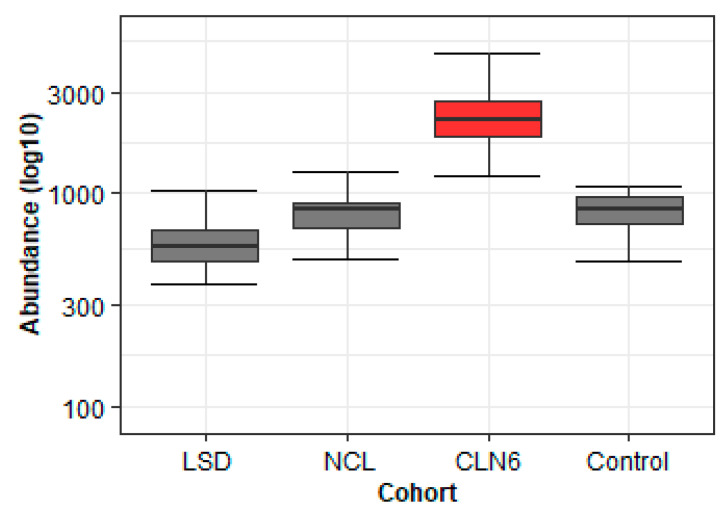
Boxplots showing the “behavior”:abundance distribution of a CLN6 (red color) specific biomarker candidate (4.42_384.3240n) in four cohorts (LSD *n* = 27, NCL *n* = 19, CLN6 *n* = 30, control *n* = 20) using methanol extraction. The circles represent the outliers. Boxes represent the 25th and 75th percentiles, while bars represent minimum and maximum values.

The reliability of the selected buffer, namely methanol–acetonitrile (3:1, *v/v*), was assessed on a full batch of 95 samples performed on the LC-MS QToF Vion. The batch included, among the CLN6 and the control group, a group made of eight types of neuronal ceroid lipofuscinoses diseases (CLN2, CLN3, CLN5, CLN7, CLN8, CLN12, and CLN14), and one that comprised ten types of LSDs (Mucopolysaccharidosis type I, Mucopolysaccharidosis type II, Mucopolysaccharidosis type IIIa, Mucopolysaccharidosis type IV, Spinal muscular atrophy, Fabry disease, Gaucher disease, Krabbe disease, Metachromatic leukodystrophy, and GM1 gangliosidosis disease). Due to the utility of this extraction, we were able to retrieve a larger number of metabolites with high intensity (over 1000), as well as identify compounds that differentiated between diseases and could be used in later stages of the discovery phase (Figure 9).

## 3. Discussion

There are numerous studies conducted on the factors that contribute to the stability of the metabolites in human samples [3]. Still, there is a big gap between the number of stability studies performed on DBS samples and non-DBS samples, such as serum, plasma, urine, and tissue [28].

In the current study, we explored the profile variation of the human metabolome using DBS from two different angles: (1) assessing the stability of metabolites over time; (2) investigating the extraction solvents suitable for metabolomics studies.

It is well known that storage conditions for a given biological material varies depending on the type of sample and the predetermined storage time. Typically, one of three temperatures is considered adequate to store blood samples: room temperature, refrigerated storage (4 °C), or freezer storage (below −20 °C) [29].

Trifonova et al. 2019 [30] examined the stability of several types of single patient-based filter cards stored for four weeks at room temperature. The obtained results showed no significant impact on DBS stability during the four-week storage period. In contrast, the study designed by Drolet et al. 2017 [22], namely a short-term stability study (maximum two weeks) of DBS and urine, showed that DBS cards are unstable at room temperature. According to our results, a limited storage time at −20 °C is crucial in maintaining the reliability of metabolomics studies, while at room temperature even short-term storage can affect the stability of the metabolites. This contradicts the study reported by Prentice et al. 2013 [31], who found that filter cards are stable for weeks at room temperature and up to a year if kept at −20 °C.

Furthermore, we observed that sample types and the card age have an impact on metabolite yield. To ensure the reliability of the data obtained from the metabolomics study, using samples prepared in an identical way and under similar storage conditions is recommended. Several studies reported on the tactics used to improve the extraction efficiencies in metabolomics [32]. Nonetheless, relatively, very few attempted to examine the global metabolome coverage of DBS, as most of them addressed either specific metabolites or other biofluids [33]. Considering the chemical diversity of the human blood metabolome, capturing all its features is challenging [34]. In principle, it is preferable to determine the extraction method that best suits a purpose and optimize it to maximize the number of metabolites. However, it is a daunting task to choose one that applies to such a large scale and fits such a broad spectrum of diseases.

Comparison of fourteen extraction methods on serum samples revealed that methanol was the most effective extraction and provided the highest metabolome coverage [35]. Similar to our results and in agreement with the previous study done by Alshammari et al. 2015 [36], methanol is a better addition to identify high-intensity metabolites. Therefore, our data highlighted the importance of the solvent on untargeted metabolomics and demonstrated that the extraction solvent for LC-MS-based metabolomics has a visible effect on biomarker-focused studies.

Although knowledge of the stability of the analytes in DBS is of crucial importance for biomarker discovery, data available on the assessment of human DBS stability for metabolomics analysis are still scarce. Existing publications on untargeted metabolomics mention several protocols intended to improve the metabolome coverage [37,38]. Yet, few targeted large cohorts of patients and, to our knowledge, none focused on disease-specific metabolites. Moreover, due to the scarce data available on the assessment of DBS applicability to metabolomics studies, the final goal is to incorporate this approach into future studies in biomarker research.

## 4. Materials and Methods

### 4.1. Chemicals and Reagents

The solvents used in metabolite extraction, such as methanol, acetonitrile, isopropanol, and formic acid 99% (eluent additive for LC-MS), were all UPLC-MS grades from Biosolve (Dieuze, France). Water LC-MS grade was purchased from VWR (Darmstadt, Germany), ammonium acetate was obtained from Sigma Aldrich (St. Louis, MO, USA), and ethanol 96% from ROTH.

### 4.2. Mass Spectrometric Analysis

The extract analysis was performed on Waters i-Class ACQUITY UPLC (Waters, Borehamwood, UK) coupled with a Vion IMS-QToF mass spectrometer (Waters, Borehamwood, UK) equipped with an ESI ion source. The chromatographic run was in the positive ionization mode in the mass range of 100–1000 *m/z*. From each extract, 10 μL was injected into a Kinetex EVO (C18, 2.1 × 150 mm, 5 μm) LC column (Phenomenex, Aschaffenburg, Germany) preheated at 50 °C with a flow rate of 0.5 mL/min. The analytes were eluted by using a linear gradient in a range from 1% to 100% B (50 mM formic acid in methanol: acetonitrile vol. 1:1) and A (50 mM formic acid in water). The following parameters were used for mass spectrometric acquisition: high-definition mass spectrometry (HDMSE), capillary voltage 1.2 kV, source temperature 150 °C, desolvation temperature 600 °C, desolvation gas 1000 L/h, cone gas 50 L/h, low collision energy 6 eV, high collision energy ramp 20–40 eV, scan mass 50–1000 *m/z*, scan time 0.5 s.

### 4.3. Data Acquisition and Analysis

The acquisition was carried out using the Unifi software v1.9 (Waters, Borehamwood, UK), and the results were exported as Unify export packages (.uep). The file was imported into the Progenesis QI software v2.3 (Nonlinear Dynamics, Newcastle upon Tyne, UK) for normalization, metabolites filtering and exported as a .csv file for statistical analysis. The analysis was conducted using MetaboAnalyst tool 4.0 [39], R version 3.6.2 [40], and the figures were produced using the package ggplot2 [41].

### 4.4. Blood Sample Collection and Preparation

In the current study, the participants were divided into two groups: (i) a control group with no LSD symptoms and DBS cards prepared in-house; (ii) an LSD group with samples (DBS card and/or EDTA blood) shipped by the physician. Following that, the LSDs samples were divided into two cohorts: one set of eighteen types of LSDs, and one of NCL disorder with its nine types of ceroid-lipofuscinosis neuronal diseases, also known as CLNs diseases (Supplementary Table S1). Blood samples derived from the control subjects were initially collected for routine metabolic research and processed within two hours of blood withdrawal. For the DBS preparation, the EDTA blood tube was gently inverted five times, and a 60 µL aliquot was spotted onto each spot of the CentoCard^®^ (Centogene GmbH, Rostock, Germany). After drying for at least four hours at room temperature, the cards were sealed into plastic bags and stored at −20 °C until further processing. The DBS samples from the LSD-affected patient were prepared by the physician and shipped to us for routine diagnostic analysis. The samples were kept at room temperature after they arrived, pending further analysis. Per analysis, five (3.2 mm diameter) center punches were taken from each card. The spots were cut with the PerkinElmer puncher (PerkinElmer LAS, Rodgau, Germany), which were then collected into a 96-well microtitration plate.

### 4.5. Dried Blood Spot Extraction

All samples were prepared under the same conditions. The extraction of the metabolites for the stability study has previously been described [30]. Briefly, an extraction mixture of 50 µL DMSO in water (3:2 *v/v*), and 100 µL internal standard 200 ng/mL (lyso-Gb2, Matreya LLC, State College, PA, USA) dissolved in ethanol was added to each well. The aforementioned protocol was modified solely for the extraction–optimization study. Here, the extraction was performed by adding 50 µL of the extraction solution (Table 1) and 100 µL internal standard solution 200 ng/mL dissolved in methanol. After adding the corresponding buffer to the DBS punches, the plate was sonicated for 10 min (Sonoswiss Ultrasonic Cleaner SW12H, Ramsen, Switzerland) and incubated for 60 min at 37 °C with 700 rpm (Heidolph, Schwabach, Germany). Following incubation, the samples were sonicated for 10 min then centrifuged for 5 min at 3500 rpm in a Hermle Z300 plate centrifuge (Hermle Labortehnik, Wehingen, Germany).

### 4.6. Patients Inclusion

The samples varied in age (children and adults), sex, time of sampling, and storage duration, thereby having considerable variations in factors that could influence the metabolome. All samples analyzed were anonymized, so there is no overall breach of data privacy. The study included 27 controls (13 male and 14 female) ranging in age from 23 to 65 years old, as well as 39 LSD patients (18 male and 21 female) ranging in age from 2 to 65 years old (Supplementary Table S2(a–c)). They were divided as follows: (1) stability study, (2) extraction study, and (3) cumulative study (Table 2).

## 5. Conclusions

The sample grouping procedure was done based on three metrics: sample types, sample age, and sample storage conditions. These key metrics are not commonly used in the existing biomarker-focused metabolomic studies. According to our findings, differences in these parameters have a considerable impact on the stability of the metabolites. In addition, the use of different solvents for the extraction has shown variable results on metabolite intensity and abundance scale. We conclude that the reliability of metabolites is higher if the following conditions are met: similar sample types, similar sample age, and similar storage conditions are obtained; methanol is used as the extraction solvent.

## Figures and Tables

**Table 1:** Summary of the amount and type of solvent used and its performance on DBS extraction.

**(a)**					
**Extraction**	**Ratio *v/v***	**Features Detected**	**Metabolites Abundance > 10.000**	**Metabolome Coverage**	**Metabolites Abundance**
dimethyl sulphoxide: water	3:2	9867	16	++++	+
isopropanol: acetonitrile: water	3:3:2	9290	49	+++	++
methanol: acetonitrile	3:1	7759	70	++	+++
ammonium acetate: water	2 mM	5970	95	+	++++
**(b)**					
**Methanol Mixtures**	**Ratio *v/v***	**Features Detected**	**Metabolites Abundance > 10.000**	**Metabolome Coverage**	**Metabolites Abundance**
methanol: acetonitrile	3:1	7759	70	+	++++
methanol	100%	9120	39	+++	+++
methanol: acetonitrile	1:1	9964	29	++++	++
methanol: water	3:1	7833	25	++	+

(+) indicates the solvents performance ranking from weakest + to strongest ++++.

**Table 2:** Distribution of the individuals by experiments.

	(1) Stability Study			(2) Extraction Study		(3) Cumulative Study		
Subjects	Control	CLN6	LSD	Control	LSD	Control	NCL	LSD
male/female	3/3	2/8	6/5	10/11	4/7	10/11	19/30	10/15
age (mean ± SD)	36.5	6.5	15.8	32	6	33	6	6.5

## Data Availability

The datasets generated and/or analyzed during the current study are available from the corresponding author on reasonable request.

## Supplementary Materials

The following are available online at https://www.mdpi.com/article/10.3390/metabo11060382/s1. Table S1: Overview of lysosomal storage diseases (LSDs); Table S2(a–c): List of the total number of patients and controls used in the study.
