# Supplementary material for: Dried Blood Spot (DBS) Methodology Study for Biomarker Discovery in Lysosomal Storage Disease (LSD)
Source: Metabolites. 2021 Jun 13;11(6):382. doi: 10.3390/metabo11060382 (PMC8231917; doi:10.3390/metabo11060382)
Supplement: Supplementary file 1 [file metabolites-11-00382-s001.zip › metabolites-1224568-Supplementary.pdf]

Supplementary Table S1. Lysosomal storage diseases (LSDs) used in the study

(*) other LSDs (lysosomal storage diseases included in the study except neuronal ceroid lipofuscinoses)

Gene	Disorder	OMIM	Enzyme deficiency / Protein defective
GLA	Fabry	301500	α-galactosidase
GBA	Gaucher	230800	β-glucocerebrosidase
GALC	Krabbe	245200	Galactoceramides
ARSA	Metachromatic leukodystrophy	25010	Arylsulfatase A
GLB1	GM1 gangliosidosis	230500	β-galactosidase
IDUA	Mucopolysaccharidosis type I (Hurler syndrome)	607015	α-L-iduronidase
IDS	Mucopolysaccharidosis type II (Hunter syndrome)	300900	Iduronate -2-sulfatase
SGSH	Mucopolysaccharidosis type IIIA (Sanfilippo syndrome)	252900	Heparan-N-sulfatase
GALNS	Mucopolysaccharidosis type IVA (Morquio syndrome)	253000	Galactose 6-sulfatase
ARSB	Mucopolysaccharidosis type VI (Maroteaux-Lamy)	253200	N-acetyl galactosamine 4-sulfatase (Arylsulfatase B)
SMN1	Spinal muscular atrophy	253300	survival Motor Neuron (SMN)
ASAH1	Farber	228000	Acid ceramidase
TTR	Transthyretin -related amyloidosis	105210	Transthyretin (Prealbumin)
FAH	Tyrosinemia type I	276700	Fumarylacetoacetate hydrolase
LIPA	Wolman	270800	Acid lipase
DMD	Duchenne muscular dystrophy	310200	Dystrophin
CFTR	Cystic fibrosis	219700	Fibrosis transmembrane conductance regulator
GAA	Glycogen storage disease II	232300	Acid alpha-1,4-glucosidase

**NCL (Neuronal ceroid lipofuscinoses)

Gene	Disease	OMIM	Encoded protein
PPT1	CLN – type 1	256730	Palmitoyl protein thioesterase 1, PPT1 soluble protein
TPP1	CLN– type 2	204500	Tripeptidyl peptidase 1, TPP1 soluble protein
CLN3	CLN– type 3	204200	CLN3 transmembrane protein
CLN5	CLN– type 5	256731	CLN5 soluble protein
CLN6	CLN– type 6	601780	CLN6 transmembrane protein
MFSD8	CLN– type 7	610951	CLN7/MFSD8 transmembrane protein
CLN8	CLN– type 8	610003	CLN8 transmembrane protein
ATP13A2	CLN– type 12	606693	CLN12/ATPase13A2 transmembrane protein
KCTD7	CLN– type 14	611726	CLN14 / Potassium channel tetramerization domain containing protein 7 soluble protein

Supplementary Table S2. List of the total number of patients and controls

(a)

Group	ID	Sex	Age
Non -LSD affected individuals (controls)	1	F	33
	2	F	35
	3	F	39
	4	F	30
	5	F	65
	6	F	23
	7	F	31
	8	F	33
	9	F	45
	10	F	32
	11	F	28
	12	F	65
	13	F	23
	14	F	32
	15	M	30
	16	M	31
	17	M	28
	18	M	41
	19	M	41
	20	M	35
	21	M	33
	22	M	30
	23	M	37
	24	M	41
	25	M	36
	26	M	28
	27	M	25

(b)

Group	ID	Gene	Sex	Age
Neuronal ceroid lipofuscinoses (NCL -LSD)	1	*ATP13A2*	F	32
	2	*CLN3*	M	8
	3	*CLN5*	M	6
	4	*CLN6*	F	5
	5	*CLN6*	F	5

	6	*CLN6*	F	6
	7	*CLN6*	F	6
	8	*CLN6*	F	7
	9	*CLN6*	F	9
	10	*CLN6*	F	45
	11	*CLN6*	F	11
	12	*CLN6*	F	8
	13	*CLN6*	F	7
	14	*CLN6*	F	5
	15	*CLN6*	M	4
	16	*CLN6*	M	5
	17	*CLN6*	M	6
	18	*CLN6*	M	8
	19	*CLN6*	M	?
	20	*CLN8*	M	8
	21	*KCTD7*	M	3
	22	*KCTD7*	M	12
	23	*MFSD8*	F	5
	24	*PPT1*	F	5
	25	*TPP1*	M	5
	26	*TPP1*	M	13

(c)

**Group**	**ID**	**Gene**	**Sex**	**Age**
**Lysosomal Storage Diseases (other LSDs)**	1	ARSB	M	6
	2	ASAH	F	1
	3	CFTR	F	0,2
	4	CFTR	F	2
	5	CFTR	M	4
	6	DMD	M	6
	7	FAH	F	24
	8	GAA	F	20
	9	GAA	F	22
	10	GAA	F	25
	11	GALC	F	3
	12	GALC	M	4
	13	GLA	M	10

	14	IDS	M	NA
	15	LIPA	F	0,3
	16	TTR	M	65
